# New Treatments for Atopic Dermatitis Targeting Skin Barrier Repair via the Regulation of FLG Expression

**DOI:** 10.3390/jcm10112506

**Published:** 2021-06-05

**Authors:** Anna Dębińska

**Affiliations:** 1st Department and Clinic of Paediatrics, Allergology and Cardiology, Wroclaw Medical University, Chałubińskiego 2a, 50-368 Wrocław, Poland; anna.debinska@umed.wroc.pl; Tel.: +48-510-066-478 or +71-770-30-91; Fax: +48-713-281-206

**Keywords:** atopic dermatitis, skin barrier, filaggrin, biologicals, small molecule therapies

## Abstract

Atopic dermatitis (AD) is one of the most common chronic, inflammatory skin disorders with a complex etiology and a broad spectrum of clinical phenotypes. Despite its high prevalence and effect on the quality of life, safe and effective systemic therapies approved for long-term management of AD are limited. A better understanding of the pathogenesis of atopic dermatitis in recent years has contributed to the development of new therapeutic approaches that target specific pathophysiological pathways. Skin barrier dysfunction and immunological abnormalities are critical in the pathogenesis of AD. Recently, the importance of the downregulation of epidermal differentiation complex (EDC) molecules caused by external and internal stimuli has been extensively emphasized. The purpose of this review is to discuss the innovations in the therapy of atopic dermatitis, including biologics, small molecule therapies, and other drugs by highlighting regulatory mechanisms of skin barrier-related molecules, such as filaggrin (FLG) as a crucial pathway implicated in AD pathogenesis.

## 1. Introduction

Atopic dermatitis (AD) is one of the most common chronic inflammatory skin disorders worldwide. It may affect people of all ages and ethnicities and is the leading cause of the global burden of skin conditions [1,2,3]. Globally, the prevalence of AD is increasing, with a lifetime prevalence of 15–30% in children and 2–10% in adults in developed countries [4,5]. AD is a highly heterogeneous disease with a complex etiology with genetic, immunological and environmental factors causing skin barrier dysfunction and dysregulation of the immune response [6,7,8,9]. It is now recognized that the interaction between those two mechanisms is crucial to the pathogenesis of AD and it determines its chronic and relapsing course [1,10,11]. Data from experimental models support the key role of genetic defects in epidermal barrier protein and highlight the importance of skin barrier dysfunction in AD pathogenesis [12,13]. There is accumulating evidence to support the “outside-to-inside” theory of AD, in which skin barrier abnormalities are the primary cause of early AD, while an adaptive immune response is induced secondary, as a downstream consequence of epidermal barrier disruption and release of barrier-driven cytokines [13,14,15,16,17,18]. Epidermal barrier abnormalities leading to impaired integrity, easy irritability, reduced water retention, increased permeability to allergens and increased susceptibility to infection have been observed in both affected and non-affected skin of patients with AD [8,9,11,19,20]. Major contributors to the epidermal barrier dysfunction in AD include reduced expression of epidermal structural proteins, altered intercellular lipid composition, imbalance in protease-protease inhibitor interactions and disordered tight junctions [19,21,22]. The downregulated expression of barrier-related proteins such as filaggrin (FLG), loricrin and involucrin is the cardinal pathologic feature of the skin that has been demonstrated in AD patients [21,22,23,24,25].

Filaggrin (FLG) is an important epidermal structural protein, crucial to the structure and function of the stratum corneum (SC). FLG and its degradation products contribute to skin hydration, pH balance, epidermal barrier integrity and microbial defense [24,25,26,27]. An increased skin permeability has been proposed as the most plausible mechanism linking FLG deficiency and AD. Proteomic analysis on skin equivalent models revealed that molecular consequences resulting directly from FLG deficiency are complex and include the dysregulation of proteins relevant to inflammatory, proteolytic and cytoskeletal functions [12]. In murine models of AD, FLG deficiency alters both the intracellular and extracellular architecture of keratinocytes, interferes with lipid secretion, reduces inflammatory thresholds to irritants and haptens, permits increased allergen penetration and enhances skin inflammation [12,13,28,29,30,31]. The immediate result of FLG deficiency in patients with AD is also a lower level of natural moisturizing factors (NMF) which drives decreased stratum corneum hydration, increased transepidermal water loss (TEWL) and xerosis [32,33]. The most widely established cause for skin abnormalities related to FLG are the loss-of-function mutations of the FLG gene that result either in a reduction in the amount of FLG protein or complete loss of FLG and its degradation products [24,34,35]. Genetic variants in the FLG gene are known to be the most significant predisposing factor for AD [36]. However, FLG mutations are only found in 10–50% of AD cases and the majority of children with AD and FLG mutations outgrow their disease [37,38]. Additionally, those carrying FLG mutations do not always develop AD [39]. On the other hand, decreased expression levels of FLG are observed in both lesional and non-lesional AD skin, regardless of FLG mutations [23,31,40]. This suggests that there are also acquired mechanisms at play that downregulate the FLG expression. According to the “outside-to-inside-and-back-outside” hypothesis, the secondary immunologic activation induces further epidermal barrier disruption, as the type 2 immunity cytokines, as well as cytokines of other T-cell subtypes, can reduce the expression of the SC proteins [14,41,42]. The level of FLG expression is also actively modulated by external stimuli and environmental stressors [24,31]. These observations suggest that FLG deficiency contributing to the development of AD may constitute one of the major targets for therapy.

Despite its high prevalence and effect on the quality of life, safe and effective systemic therapies approved for long-term management of AD are limited. One of the goals of the current research efforts is the development of new therapeutic approaches that target specific pathophysiological pathways. Based on the aforementioned insights into the role of the FLG in the pathogenesis of AD, restoring skin barrier function through upregulation of FLG expression could be a potential therapy for all patients with AD regardless of mutation status. The purpose of this review is to highlight fundamental regulatory mechanisms of skin barrier-related molecules, such as FLG, and to discuss innovations in the therapy of AD, including biologics, small molecules therapies and other drugs targeting FLG upregulation (Table 1).

## 2. Genetic Causes of FLG Deficiency

The FLG gene is a large repetitive gene located in the epidermal differentiation complex (EDC), a cluster of more than 70 genes located on chromosome 1q21 [43,44]. The EDC region includes genes encoding many barrier-related proteins that are essential for epidermal maturation and differentiation [43,44]. The FLG mutations are located in the third exon of the gene and as the loss-of-function mutations they cause a reduction or complete absence of the expressed protein depending on the number of mutations the individual carries [35,45]. An impressive series of replication studies that have followed the initial publication by Palmer et al. showed that FLG loss-of-function mutations are the most significant genetic risk factor associated with AD [36,37,46,47]. Currently, more than 500 nonsense mutations in FLG have been identified in different ethnic populations with race-specific prevalence [35]. One in ten Europeans carries at least one FLG loss-of-function mutation and the prevalence of FLG mutations in Asian subjects reaches 3% to 6% [48,49,50]. Although it was previously reported that FLG mutations are less common in African populations, recent whole-exome sequencing studies have revealed rare FLG variants in this ethnic group [51,52]. Additionally, the spectrum of the most frequent mutations varies between Asian populations, African populations and white populations [48,51,53]. Nevertheless, several genome-wide association studies (GWASs) that include meta-analyses were able to replicate the association of FLG with AD in different populations [47]. Thus, FLG loss-of-function mutations remain the widely replicated gene in AD, with the overall ratio estimated to range from 3.12 to 4.78 [36]. Moreover, FLG loss-of-function mutations predispose to a distinct phenotype of AD including palmar hyperlinearity, earlier onset, protracted and more severe course, increased risk of allergic sensitization, asthma and contact allergy, and higher infection susceptibility [24,54,55]. In addition to loss-of-function mutations intragenic copy number variation (CNV) within FLG, with alleles encoding 10, 11 and 12 FLG monomers repeats may also result in decreased levels of FLG expression [56]. The large-scale study indicated that the lower copy number within FLG exon 3 contributes to the risk of AD and its severity. Interestingly this effect was independent of classic loss-of-function mutations in FLG [56,57,58].

Therefore, the development of a therapy aimed to treat the most significant genetic defect in AD is warranted. One of the theoretical gene-based approaches to FLG replacement might include the use of “read-through” drugs which focusing on mutant allele and might be achieved by skipping of the nonsense FLG mutations during RNA splicing or incorporating of amino acids at the mutation site. These drugs are currently being tested for other genetic diseases with promising results [59,60]. Another potential approach might be the use of drugs increasing FLG expression by manipulating the genetic regulatory element and the promotor of the healthy alleles. Despite being potentially applicable, therapies directly targeting the reduced production of FLG protein due to genetic variation are not currently available. However, the development of “read-through” drugs for the treatment of ichthyosis vulgaris and atopic conditions is patented.

## 3. Direct FLG Replacement Therapy

Direct FLG replacement via topical applications remains a challenge but still a potentially attractive approach [18]. However, many obstacles must be overcome to deliver an exceptionally large protein such as FLG to the keratinocyte cytoplasmic space, in the relevant differentiation-specific compartments [61]. In a proof-of-concept study, Stout et al. obtained promising results with the topical application of an engineered FLG monomer linked to a cell-penetrating peptide in cell culture, skin equivalents and mouse models [62]. Topically applied functional FLG monomers are able to penetrate epidermal tissue, be internalized into the appropriate cell type, and be processed to a size similar to wild-type functional barrier peptides to restore necessary barrier function [62]. These results suggest that topical application of targeted recombinant partial FLG protein may be an applicable and useful therapeutic approach, but further work to determine the safety and efficacy in human subjects is required.

## 4. Indirect FLG Replacement Therapy

On the skin surface, FLG is deiminated and degraded to release its constituent amino acids and their metabolites such as trans-urocanic acid (UCA) and pyrrolidine carboxylic acid (PCA) [26,27]. These FLG degradation products, together with other hydrophilic components such as urea and lactic acid, form the NMF that is crucial for the maintenance of epidermal homeostasis [25,27]. It was demonstrated that the SC levels of PCA, UCA and histidine are influenced by both FLG genotype and the severity of AD [42]. Thus, FLG replacement by topical application of its metabolites constitutes another potential target for future novel treatments. Peltonen et al. evaluated efficacy, safety and tolerability of topical cis-urocanic acid (cis-UCA) cream in three randomized phase 1/2b clinical trials in patients with mild-to-moderate AD. The treatment with 5% cis-UCA emulsion cream was well tolerated and has been found to reduce TEWL and cutaneous erythema as well as to improve the Eczema Area and Severity Index (EASI) score and physician’s global assessment [63]. FLG is a histidine-rich protein and histidine is a substrate for histidase which generates UCA in upper SC [26,27]. In vitro studies confirmed that L-histidine significantly increased FLG production and improved the skin barrier function in human skin-equivalent models [64]. Those findings point to the possibility of using oral L-histidine supplementation in the therapy of AD. Data from the clinical pilot study indicated that once-daily oral L-histidine significantly reduced signs and symptoms of AD in adults with SCORAD (Scoring Atopic Dermatitis) reducing by 34% and 32% over 4 and 8 weeks of treatment, respectively. This effect was similar to that found for mid-potency topical corticosteroids (TCs) [64]. Recently, a survey of adults with AD taking 4 g L-histidine daily reiterated the lack of causal adverse events and also reported a 33% reduction in topical corticosteroids use [65]. A placebo-controlled, clinical pilot study conducted in young children with AD taking 0.8 g L-histidine daily, showed that eczema area and severity index scores were reduced by 49% at 12 weeks, whereas the effect was not observed in the placebo group [65]. Those studies indicate that oral L-histidine supplementation is a safe, nonsteroidal approach suitable for long-term use in skin conditions associated with FLG deficits, such as AD.

## 5. Acquired FLG Deficiency–Regulation of FLG Expression

Accumulated data have shown that FLG expression is not stable and is amenable to modulation by external and internal stimulants [24,31,66,67,68]. The downregulation of FLG is confirmed to be systemic and secondary to dysregulated expression of Th2-associated (IL-4, IL-13) and Th22-associated (IL-22) cytokines observed in the skin of AD patients [9,11,41,42,66,69]. It has been also suggested that IL-20, IL-24, IL-25, IL-31 and IL-33 can potentially contribute to FLG deficiency, although the mechanisms involved are not fully understood [70,71,72,73,74,75,76] (Table 2). On the other hand, recent studies reported that activation of aryl hydrocarbon receptor (AHR), a ligand-activated transcription factor, plays an essential role in upregulating the expression of FLG [66,67,68]. There are also findings available about environmental factors and exposomal influences that have a significant effect on FLG expression [24,31]. 

Therefore, therapeutic strategies that involve blocking the cytokine-mediated FLG downregulation or enhancing FLG expression may be beneficial in treating AD. A primary target is downstream of the IL-4/IL-13 axis and the AHR axis, while the other minor targets, although promising, are not fully understood. 

### 5.1. Upregulation of FLG Expression by AHR Activation

**Figure 1:**
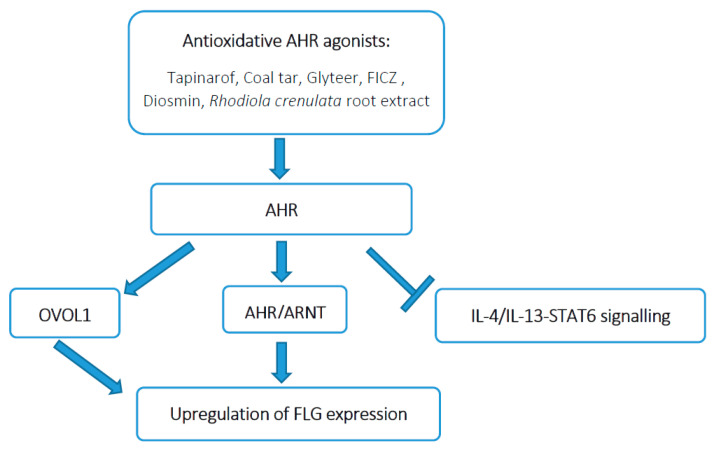
Simplified scheme of upregulation of filaggrin (FLG) by the aryl hydrocarbon receptor (AHR) axis. AHR activation upregulates FLG expression by direct activation of AHR/AHR-nuclear translocator (ARNT)/FLG gene promoter and upregulation of OVOL-like 1 (OVOL1) transcription factor signaling. Additionally, AHR activation inhibits the IL-4/IL-13-mediated signal transducer and activator of transcription (STAT)6 signaling and restores FLG expression. FICZ: The 6-Formylindolo[3,2-b]carbazole.

The aryl hydrocarbon receptor (AHR) is a ligand-activated transcription factor expressed in epidermal keratinocyte that is essential to the coordinated upregulation of the EDC gene, including FLG [67,77,78,79,80]. As a chemical sensor AHR is activated by small-molecule, xenobiotic chemicals of exogenous and endogenous origin, including dioxins, phytochemicals, microbial bioproducts, and tryptophan photoproducts [68,78,79,81,82,83,84,85,86,87,88]. Both oxidative (dioxins and bezno[α]pyrene) and antioxidative (coal tar, soybean tar, phytochemicals) AHR ligands induce upregulation of FLG and other differentiation-related molecules [68,87,88,89,90]. After ligand binding, cytoplasmic AHR translocates into the nucleus where it dimerizes with AHR-nuclear translocator (ARNT), binds DNA and upregulates transcription of targeted genes [77,91]. Thus, AHR activation promotes FLG expression directly via AHR/ARNT binding to the one or two xenobiotic responsive elements in the promoter region of the FLG gene [79,80,87]. In addition to this pathway, the AHR signaling upregulates the expression OVO-like 1 (OVOL1) transcription factor that after its translocation into the nucleus binds EDC and upregulates FLG [67,68,88,92]. It was demonstrated that OVOL1 levels correlate with FLG mRNA and protein levels and that AHR-mediated FLG upregulation is abrogated in OVOL1-knockdown NHEKs [67]. On the other hand, IL-4 and IL-13 consistently inhibit FLG expression by interfering with the OVOL1 nuclear translocation [67,93]. However, recent studies have clearly shown that suitable AHR activation rescues Th2 cytokine-mediated FLG downregulation [93,94,95] (Figure 1). In parallel, AHR signaling regulates oxidative stress in keratinocytes. Oxidative AHR ligands induce the generation of reactive oxygen species (ROS) leading to DNA damage and inflammation [81,87]. However, antioxidative AHR ligands inhibit ROS generation via activation of nuclear factor-erythroid 2-related factor-2 (NRF2), which is a master switch for antioxidative signaling [68,85,86,94]. Therefore, antioxidative AHR agonists are expected to be promising candidates for AD treatment in which skin barrier disruption, Th2 inflammation and oxidative stress upregulation are observed.

The topical application of coal tar is the oldest known dermatological treatment and is efficacious in reducing inflammation and itch in AD. Coal tar consists of a wide range of polycyclic aromatic hydrocarbons [89,94]. Van den Bogaard et al. first demonstrated that coal tar activates AHR, upregulates FLG expression to wild-type levels, and restores the IL-4/IL-13-STAT6-mediated downregulation of FLG [89]. Glyteer is a delipidated soybean tar that has been widely used for the treatment of various inflammatory skin diseases in Japan since 1924 as an alternative to a coal tar remedy. Glyteer exhibits biological properties similar to those of coal tar [90]. Coal tar and glyteer are crude mixtures of various chemical compounds with not consistent therapeutic activity, thus further scientific and clinical studies of them have not been conducted. However, the randomized controlled pilot study comparing the efficacy of topical coal tar to TCs in children aged 1 to 16 years with AD has recently been completed but results are not available for review. The 6-Formylindolo[3,2-b]carbazole (FICZ), a tryptophan photoproduct generated by UVB exposure, is a high-affinity ligand for AHR that itself activates the AHR-CYP1A1 pathway, generates ROS and upregulates FLG expression [83]. In the murine dermatitis model, topically applied FICZ significantly increased FLG expression, improved dermatitis clinical scores and TEWL and inhibited AD-like skin inflammation with a decrease of IL-22 expression [96]. Recently, *Rhodiola crenulata* root extract (RCE) that contains several AHR agonists including luteolin, quercitrin, and isoquercitrin has been also shown to upregulate FLG and LOR expression in an AHR-OVOL1-dependent manner [97]. Diosmin is another natural-derived AHR agonist whose effects on skin barrier restoration have recently been investigated. Diosmin upregulated FLG expression and reversed the Th2 cytokine-mediated downregulation of FLG protein level in normal human epidermal keratinocytes. In the human skin equivalent models treatment with diosmin also increased epidermal thickness [95].

Tapinarof (or benvitimod), a single molecular high-affinity AHR agonists, is a naturally derived hydroxylated stilbene that showed promising efficacy as a topical treatment for AD [94,98]. Tapinarof activates both AHR and NRF2, resulting in increased skin barrier protein expression, including FLG, reduced oxidative stress, decreased proinflammatory Th2 cytokine expression and re-established skin homeostasis [98,99]. Two early clinical trials demonstrated that topical tapinarof at 0.5% and 1.0% is efficacious for the treatment in patients with mild-moderate and severe AD. Gradual improvement in IGA (Investigator Global Assessment), EASI, and SCORAD and in pruritus for patients randomized to tapinafor was observed [100,101]. In phase 2, double-blind, vehicle-controlled trial in patients with AD aged 12 to 65 years, tapinafor achieved a 75% or greater improvement in EASI score (EASI-75) at week 12 in more than 50% of patients treated once daily. Treatment success was maintained for 4 weeks after the end of treatment with tapinarof [102]. More recently, another phase 2b trial demonstrated that 1% tapinarof cream led to statistically significant and clinically meaningful improvements in efficacy analyses, including the proportion of patients achieving EASI-75 and EASI-90 (90% or greater improvement in EASI score) and the overall improvements in EASI scores and BSA (body surface area) affected. In addition, a significantly greater proportion of patients treated with tapinarof cream rated AD and pruritus symptom severity as very or moderately improved, and POEM (patient-oriented eczema measure) improvements were observed in all groups [103] (Table 3). These results support the hypothesis that tapinarof, as a nonsteroidal topical agent, represents an important advance in the development of topical treatment for AD and warrants further study in phase 3 clinical trials.

### 5.2. Downregulation of FLG Expression by IL-4/IL-13

Historically, it has been demonstrated that interleukin IL-4 and IL-13 play a key role in AD pathogenesis [9,11,69,104]. The gene expression of Th2-derived cytokines, IL-4 and IL-13, is significantly higher in the lesional skin of AD patients compared with the normal skin or unaffected AD skin [104,105,106]. The Th2 predominance is likely to progress from non-lesional to lesional skin and from acute and chronic lesions in AD [107]. The importance of Th2 deviations in AD is supported by the fact that AD-like phenotype can be induced in murine models overexpressing IL-4 and IL-13 [108,109,110]. Moreover, AD has been shown to be associated with IL-4 and IL-13 polymorphisms [111,112,113]. Notably, IL-4 and IL-13 not only lead to the recruitment of inflammatory cells but also are known to compromise the integrity of the skin barrier by downregulating the expression of FLG [66,93]. Howell et al. demonstrated that keratinocytes differentiated in the presence of IL-4 and IL-13 exhibited significantly reduced FLG gene expression, even in patients without FLG mutations, confirming an acquired FLG deficiency that can be modulated by the inflammatory response [41]. Keratinocytes express IL-4Rα/IL-13Rα1 receptor complex shared by both IL-4 and IL-13 [113]. Binding of IL-4 and IL-13 to IL-4Rα/IL-13Rα1 complex activates Janus kinase 1 (JAK1), JAK2 and tyrosine kinase 2 (TYK2) and subsequently induce the phosphorylation of signal transducer and activator of transcription (STAT)6 and STAT3 [66,114,115]. IL-4/IL-13-mediated activation of STAT6 and STAT3 pathway downregulates the expression of FLG, disrupts the skin barrier function and increases the production of alarmins such as IL-25, IL-33 and thymic stromal lymphopoietin (TSLP) [9,11,66]. In addition, IL-4 and IL-13 downregulate FLG expression by interfering with OVOL1 signaling. IL-4 and IL-13 inhibit its cytoplasmic-to-nuclear translocation and thus reduce the FLG expression [67,93]. Furthermore, IL-4/IL-13-mediated STAT6 activation upregulates periostin and subsequently enhances IL-24 production [92,116]. Therefore, all of the aforementioned mechanisms by which IL-4 and IL-13 reduce FLG expression form the basis of a novel and promising therapeutic approach.

#### 5.2.1. IL-4/IL-13 Inhibitors

Dupilumab is the first biologic approved as a first-line treatment for moderate-to-severe atopic dermatitis in patients aged 6 years and older in the USA and in patients aged 12 years and older in the EU (approval for children aged 6–12 years is pending). Dupilumab is a fully human monoclonal antibody that inhibits both IL-4 and IL-13 signaling pathways by blocking their shared IL-4Rα subunit [117,118]. Translational studies demonstrated that dupilumab reduces the expression of Th2-associated cytokines and chemokines as well as Th17/Th22 immunity markers. Additionally, after 4 weeks of dupilumab treatment, a significant reduction in the expression of the epidermal hyperplasia-related gene, T cells and dendritic cells were observed [119,120]. Concurrent with these changes, dupilumab increases the expression of terminal differentiation genes such as FLG [120].

The efficacy of dupilumab has been studied in several phase 3 trials that demonstrated efficacy and a favorable safety profile in patients with moderate-to-severe AD inadequately controlled with topical medications. In SOLO1 and SOLO2 trials dupilumab monotherapy in adults with AD demonstrated significant improvements in disease severity as measured by the EASI and Investigator’s Global Assessment. At week 16, up to 52% of patients receiving dupilumab achieved EASI-75 compared to 15% of placebo. Patients also reported improvements in clinical symptoms, pruritus, anxiety and depression. Additionally, significantly more patients in dupilumab groups had improvement in Dermatology Life Quality Index (DLQI) score [121]. In another phase 3 study (LIBERTY AD CHRONOS) investigating long-term efficacy in adults, dupilumab combined with standard TCs treatment showed an improved disease activity for a period of 52 weeks with acceptable safety [122]. The CAFÉ study, another phase 3 study, presented effectiveness in a potentially highly refractory patient population with the most previously treated cyclosporine A (CsA) [123]. Importantly, observations from large real-world patient populations for dupilumab largely accord with trial data. A recent review of real-life data from 22 studies, presented dupilumab as a successful and well-tolerated therapy for AD that demonstrated a significant reduction in EASI score as well as clinical improvement along with the quality of life improvement [124]. Dupilumab performed favorably in phase 3 trials in pediatric patients. In two recent trials focused on adolescents aged between 12 and 18 years (LIBERTY AD ADOL) and children aged between 6 and 12 years (LIBERTY AD PEDS), dupilumab showed long-term safety and efficacy also in those age groups [125,126]. Many trials with dupilumab in the pediatric population are ongoing, including evaluation of the efficacy in patients aged 6 months to 17 years, and characterizing long-term safety and efficacy with long-term use [127] (Table 3).

Dupilumab consistently demonstrates an acceptable, placebo-like safety profile, with conjunctivitis, nasopharyngitis and injection-site reactions as the most common adverse events. Data from three randomized phase 3 trials supported the use of dupilumab as a systemic treatment for the long-term management of moderate-to-severe AD without routine laboratory monitoring in clinical practice [121,122,123]. In a follow-up study in adults given 300 mg dupilumab weekly up to 76 weeks, Deluren et al. showed sustained efficacy in 92.9% of patients with no additional safety signals [128]. However, further study should focus on long-term and uncommon side-effects that are insufficiently assessed in trials to date.

#### 5.2.2. IL-13 Inhibitors

AD has been always considered as a paradigmatic in which both IL-4 and IL-13 play pivotal roles, however, recent evidence confirmed that these cytokines are differentially expressed and have different functions in atopic inflammation [8,129,130]. IL-13 is likely preferentially to participate in the peripheral inflammatory response, at the tissue level, including the skin [130,131]. Tissue-residing ILC2 (type 2 innate lymphoid cell) have been reported to expand and produce IL-13, but not IL-4, when they are activated by the keratinocyte-derived alarmins [130,132,133]. Recent transcriptomic analyses have revealed that in AD, IL-13 is expressed at a high level in both subacute and chronic skin lesions, whereas IL-4 expression is low or nearly undetectable [134]. Additionally, expression levels of IL-13 in lesional skin have been strongly correlated with disease severity, as measured by the SCORing atopic dermatitis tool [135]. The overexpression of IL-13 causes skin barrier dysfunction by decreasing the FLG expression via down-regulation of the OVOL1-FLG axis and up-regulation of periostin-IL-24 axis [92]. Since IL-13 is considered to be the primary cytokine involved in AD inflammation, biologics specifically targeting the IL-13 cytokine alone may offer an important potential therapeutic approach.

Lebrikizumab is a monoclonal antibody that inhibits IL-13 signaling by binding IL-13 and preventing heterodimerization of IL 13*α*1/IL-4R*α* [136]. In a proof-of-concept phase 2 study of adults with moderate-to-severe AD, lebrikizumab was investigated as an add-on therapy to TCs. The primary endpoint, a 50% reduction in EASI score (EASI-50) at week 12, was achieved in a significantly large number of patients with lebrikizumab than with placebo (82.4% in the treatment group vs. 62.3% in the placebo group) [137]. In a recent phase 2b trial of lebrikizumab monotherapy a significant dose- and frequency-dependent improvement in EASI score was observed in adult moderate-to-severe AD patients [138]. Lebrikizumab was well tolerated with most common adverse events such as upper respiratory tract infections, conjunctivitis and herpesvirus infections that were mild and occurred at a similar incidence in lebrikizumab groups compared to placebo [138]. Lebrikizumab is currently in phase 3 trials in adults and children with AD aged 12 years and more [127] (Table 3).

Tralokinumab is a fully human IgG4 antibody with a mode of action different from lebrikizumab. Tralokizumab specifically binds to IL-13, preventing this cytokine from binding to both IL-13Rα1 and IL-13Rα2 [139]. Tralokinumab studied in different doses in phase 2b study in adults with moderate-to-severe AD showed a significant improvement in EASI and IGA scores. Substantially better efficacy was observed particularly in patients with an elevated level of IL-13 related biomarkers, DDP-4 (dipeptidyl peptidase-4), and periostin [140]. This indicates that tralokinumab may be a suitable candidate for biomarker-based personalized therapy in the future. Recently Wollenberg et al. reported the results of the first phase 3 trials (ECZTRA1 and ECZTRA2) to investigate the long-term efficacy and safety of tralokinumab monotherapy in adult patients with moderate-to-severe AD for up to 1 year. Tralokinumab demonstrated superiority over placebo during 16 weeks of treatment across multiple outcome measures reflecting the signs and symptoms of AD, including itch and sleep scores. Long-term observation up to 52 weeks confirmed efficacy and safety with mild or moderate conjunctivitis as adverse events [141]. The ECZTRA 3 study evaluated the efficacy and safety of tralokinumab in combination with TCs in AD, which is more reflective of the likely clinical use in daily practice [142]. The use of tralokinumab in pediatric AD is currently being studied in two phase 3 clinical trials [127]. Other biologic such as ASLAN004, a fully human monoclonal antibody that targets the IL-13Rα1 receptor, is currently in clinical trials for the treatment of AD [130] (Table 3).

#### 5.2.3. JAK Inhibitors

The Janus kinases (JAKs), a family of tyrosine kinases (TYKs), including JAK1, JAK2, JAK3, and TYK2, are the first signal transducers in a pathway from the cell membrane to the nucleus. They are associated with the intracellular domain of the cytokine receptors, after the binding of ligands to receptors, they are phosphorylated and transfer the signal to STAT family transcription factors which subsequently translocate to the nucleus leading to the activation of targeted gene expression [143]. The JAK-STAT pathway is a master regulator of immune function and has been implicated in modulating multiple immune pathways involved in AD, including Th2, Th22, Th1, and Th17 [144,145]. Several studies have demonstrated significant overexpression of JAKs and activation of JAK-STAT signaling within lesional skin of AD patients [146,147]. Moreover, mice harboring point mutation leading to JAK1-specific hyperactivation develop spontaneous skin barrier disruption and pruritic dermatitis [148]. In AD, JAK-STAT signaling induces Th2 cytokines and consequently downregulates the expression of skin barrier-related proteins such as FLG. Additionally, JAK-STAT pathways are involved in eosinophil activation, B-cell maturation, up-regulation of epidermal chemokines, and down-regulation of antimicrobial peptides [149,150]. Thus, inhibiting JAKs that regulate multiple steps in AD pathogenesis seems to be conceptually attractive as a treatment option [151,152,153].

Recent studies on spontaneous and induced rodent AD models have demonstrated that JAK inhibitors reduced skin inflammation, as evidenced by decreased levels of proinflammatory Th2 cytokines in the skin and IgE in serum. In addition, JAK inhibitors reduced the skin severity score and itch behavior in AD mice [154,155,156]. Since IL-4 and IL-13 affect keratinocyte differentiation and inhibit the expression of FLG through JAK-STAT signaling, JAK inhibitors potently restore FLG expression following in vitro pretreatment with IL-4/IL-13 cytokine in human keratinocyte and improve skin barrier function [157,158]. In nonclinical studies, topical application of pan-JAK inhibitor, JTE-052, reduced skin inflammation and improved skin barrier function, permitting increases in FLG expression and NMF levels in a murine model of AD and dry skin. Moreover, JTE-052 promoted the production of FLG and NMFs in an experimental human skin graft model. Interestingly, in normal human keratinocytes and in the reconstructed human skin equivalent model JTE-052 promoted the production of terminal differentiation proteins, including FLG in the presence or absence of Th2 cytokines by inhibiting STAT3 activation [157]. Clarysse et al. have shown that tofacitinib (JAK1/3 inhibitor) pretreatment preserved epidermal morphology, reduced STAT3 and STAT6 phosphorylation and upregulated FLG gene expression in 3D skin models of AD [158]. Recent experimental studies on human keratinocytes have demonstrated that CYT389, a novel JAK2 inhibitor, restored FLG expression and alleviated IL-13 induced skin barrier damage via targeting IL-13Rα1 and STAT3 [159]. These findings demonstrate the feasibility of JAK inhibitors as possible therapeutic agents for AD that work by improving skin barrier function. Based on promising results from preclinical models, JAK inhibitors are currently being studied as systemic and topical therapeutics for AD.

Delgocitinib (JTE-052) is the topical pan-JAK inhibitor, which has inhibitory effects on all types of JAK family kinase [160]. It is the only JAK inhibitor currently approved as a topical agent for the treatment of moderate to severe AD. In phase 3, double-blind, vehicle-controlled studies, delgocitinib 0.5% ointment demonstrated remarkable improvement of EASI over 4 weeks and this effect was sustained through the following 24-weeks extensional treatment period with mild adverse events [161]. In addition, the long-term safety and efficacy of delgocitinib ointment were reported in a 52-week open-label study of Japanese adult patients with AD. The most common adverse effects included nasopharyngitis, contact dermatitis, acne, and application site folliculitis [162]. Delgocitinib ointment improved clinical signs and symptoms also in pediatric patients aged 2 to 15 years with AD and was well tolerated [163]. Delgocitinib has currently finished its phase 3 clinical trials in both children and adults and was approved for the treatment of AD in Japan in 2020. Tofacitinib, a potent JAK1/JAK 3 inhibitor, is also under development as a topical treatment agent for AD. A phase 2a trial in adults with moderate-to-severe AD showed a large reduction of pruritus by 2 days and significant improvement in EASI score by 4 weeks [164]. Ruxolitinib is another topical selective inhibitor of JAK-1 and JAK-2. In a phase 2 trial, ruxolitinib showed significant efficacy in EASI score and provided a clinically meaningful reduction in itch and QoL (Quality of Life) burden [165]. The top-line results detailed in a presentation of two phase 3 trials of ruxolitinib revealed its superior efficacy in IGA-TS, EASI-75, and a ≥4-point reduction in itch NRS score and application of ruxolitinib cream brought about rapid (within 12 h of initiation of therapy), substantial, and sustained reduction in itch [166]. Currently, a phase 1 study in children aged 2 to 17 years and two phase 3 studies in patients aged 12 years and older is underway [167]. Additional JAK inhibitors such as cerdulatinib (RTV-502) and SNA-125 are currently being investigated in a phase 1/2a clinical trial for use in AD, however, no data is currently available for review (Table 3).

In addition, several JAK inhibitors are under development for AD treatment via oral administration. Baricitinib is an oral JAK1/JAK2 inhibitor approved for the systemic treatment of rheumatoid arthritis. As two phase 3 clinical trials for baricitinib in AD are currently completed, it may be among the first JAK inhibitors to be approved for systemic AD treatment. Those phase 3 monotherapy trials, BREEZE-AD1 and BREEZE-AD2, confirmed significant clinical efficacy in both baricitinib doses of 2 mg and 4 mg with a good safety profile for patients with moderate-to-severe AD who had an inadequate response to topical therapies. The improvement in itch was achieved as early as week 1 for 4 mg and week 2 for 2 mg [168]. Preliminary data were recently released for a phase 3 trial (BREEZE-AD4) that assessed the efficacy and safety of baricitinib in combination with topical corticosteroids in patients with moderate-to-severe AD and contraindications to or failure of therapy with CIs (calcineurin inhibitors) [169]. Current ongoing phase 3 trials for baricitinib are mainly focusing on long-term effectiveness and the possible use in children and adolescents. Upadacitinib is a second-generation selective JAK1 inhibitor and it is a promising therapy not only for AD but also for additional inflammatory diseases, with 37 clinical trials currently underway evaluating its use in various disorders. A phase 2b trial in adults AD reported a dose-response relationship for upadacitinib efficacy as the 30 mg once daily dose was associated with the most significant reduction in EASI and appeared to present the best benefit/risk profile [170]. Currently, a phase 3 study including younger patients with AD is underway. Abrocitinib is another selective oral JAK1 inhibitor. In a phase 2b study abrocitinib taken once daily showed dose-dependent improvement in EASI score achieved by 82.6% of patients in the 200 mg abrocitinib group and 59% of patients receiving 100 mg of abrocitinib as well as improvement in IGA score at the week 12 [171]. Recently, two phase 3 trials in patients aged 12 years or older with moderate-to-severe AD showed that abrocitinib succeeded in clinical effectiveness and rapid onset of action with good tolerability and no unexpected safety events [172,173]. Other phase 3 trials with long-term treatment periods are now being carried out. Gusacitinib (ASN002) is a potent, dual inhibitor of pan-JAK and SYK kinases. In the phase 1b study, gusacitinib showed strong clinical improvement and rapid onset of action with associated improvements in systemic inflammation, including significant downregulation of several serum biomarkers involved in Th1, Th2 and Th17/Th22 immunity [174]. There is a phase 2 extension study to evaluate the long-term safety, tolerability, and efficacy of gusacitinib in subjects with moderate to severe AD up to 2021. The top-line press released results regarding the phase 2b study (RADIANT) reported that gusacitinib showed a rapid and statistically significant reduction in pruritus as well as a statistically significant reduction in EASI score from baseline in moderate-to-severe AD patients [175] (Table 3).

To sum up, JAK inhibitors demonstrate considerable efficacy for the treatment of AD. However, more studies should be conducted to evaluate their long-term efficacy and safety profile in particular. Involvement of the JAK-STAT pathway in the signaling of multiple cytokines, mediating immune response and hematopoiesis suggests a potentially increased risk of infection, thromboembolic events and hematological events [143,150]. The most current data from clinical trials on the use of JAK inhibitor in AD suggest that headaches, nausea, and mild-to-moderate infections, mainly nasopharyngitis, are the most commonly reported adverse effects [163,172,173]. The overall incidence of serious adverse effects of using oral JAK inhibitors for AD treatment is low, however, a topical formulation of those drugs appears to have a more favorable safety profile.

### 5.3. Downregulation of FLG Expression by IL-22

In addition to Th2 deviation, IL-22 produced by Th22 cells is also linked to the chronicity and amplification of skin inflammation in AD [69,104,105]. IL-22 is significantly increased in AD lesions and it correlates with disease severity [104,176]. IL-22 signaling occurs via a heterodimeric receptor composed of IL-22R1 and IL-10R2 and activates JAK1/TYK2-STAT3 pathway in keratinocytes [177]. IL-22 overexpression promotes epidermal proliferation and disrupts barrier function by inhibiting the terminal differentiation of keratinocyte. IL-22 inhibits the expression of FLG and also upregulates the IL-24 expression, which may reduce FLG expression via JAK1-STAT3 activation [70,178,179]. Thus, there is a strong rationale for anti-IL-22 therapy in AD patients. This treatment seems to be particularly promising amongst African American, Asian and pediatric patients with AD, who are characterized by dominant Th22 polarization and present a Th17/Th22 endotype [54].

Fezakinumab is an IL-22-blocking monoclonal antibody that, in phase 2a study in adult patients with AD, showed significant clinical improvement versus placebo. However, the result was observed only in patients with severe disease (SCORAD > 50), whereas there was no significance in reducing the SCORAD score in patients with milder disease [180] (Table 3). In another study of fezakinumab transcriptomic improvement and downregulations of multiple immune pathways, including Th1, Th2, Th17, and Th22, were restricted to the subgroup of patients with baseline IL-22-expression [181]. Thus, fezakinumab may be a potential candidate for a personalized medicine approach in AD.

**Table 3:** Novel topical and systemic targeted therapies of AD.

Agent	Target(s)	Type of Formulation	Phase	Past Clinical Trial Results	Most Common Side Effects
Tapinarof	AHR	Topical	2	Improvement in EASI, SCORAD, IGA, pruritus scores and reduction in affected BSA [100,101]	No serious side effects
			2	Improvement in EASI-75, IGA and itch scores, 1% was the most effective [102]	Nasopharyngitis, Folliculitis
			2	Improvement in EASI-75, EASI-90, IGA, pruritus and POEM scores and reduction in affected BSA [103]	No serious side effects
Dupilumab	IL-4Rα	Oral	3	SOLO1 and SOLO2: Improvement in EASI-75 and IGA scores, as well as in patient-reported outcomes (PROs), symptoms of anxiety/depression, pruritus and DLQI [121]	Injection-site reactions and conjunctivitis
			3	LIBERTY AD CHRONOS: Improvement in EASI and IGA scores for a period of 52 weeks [122]	No significant dupilumab-induced laboratory abnormalities were noted
			3	LIBERTY AD PEDS: Improvement in IGA, EASI-75, itch scores and QoL in children aged 6–11 years with severe AD [125]	Injection-site reactions and conjunctivitis
			3	LIBERTY AD ADOL: Improvement in IGA and EASI-75 scores in adolescents aged 12–18 years [126]	Injection-site reactions and conjunctivitis
Lebrikizumab	IL-13	Oral	2	TRIBLE: Improvement in EASI-50, SCORAD and pruritus for dose 125 mg every 4 weeks [137]	No serious side effects
			2	Dose-dependent, significant improvement in EASI, IGA, in the pruritus numeric rating scale (NRS) scores [138]	Upper respiratory tract infections, conjunctivitis and herpesvirus infections
Tralokinumab	IL-13	Oral	2	Improvement in EASI and IGA, SCORAD, DLQI scores and pruritus numeric rating scale for dose 300 mg, better efficacy in patients with elevated DDP-4 and periostin [140]	Upper respiratory tract infections
			3	ECZTRA1 and ECZTRA2: Improvement in pruritus, sleep interference, DLQI, SCORAD and Patient-Oriented Eczema Measure, monotherapy was effective and was well tolerated up to 52 weeks [141]	Upper respiratory tract infections and conjunctivitis
Fezakinumab	IL-22	Intravenous	2	Improvement in SCORAD, IGA and BSA [180]	Upper respiratory tract infections
Delgocitinib	Pan-JAK (JAK1, JAK2, JAK3, TYK2)	Topical	3	Improvement in EASI for 0.5% ointment, very rapid improvement of pruritus [161,162]	No serious side effects
			2	Improvement in EASI score in pediatric patients aged 2–15 years [163]	No serious side effects
Tofacitinib	Pan-JAK (more selective for JAK1 and JAK3)	Topical	2	Significantly decreased EASI, PGA, BSA by 4 weeks and pruritus by 2 days [164]	No serious side effects
Ruxolitinib	Selective JAK1, JAK2	Topical	2	Dose-dependent improvement in EASI, IGA, QoL scores and rapid itch reduction. 1.5% was the most effective [165]	No serious sideeffects
			3	Superior efficacy in IGA-TS, EASI-75, and rapid and sustained reduction in itch NRS score [166]	No serious side effects
Baricitinib	Selective JAK1, JAK2	Oral	3	BREEZE-AD1 and BREEZE-AD2: Improvements in IGA, EASI, itch scores and in night-time awakenings, skin pain and QoL for both 2 mg and 4 mg doses [168]	Nasopharyngitis and headache
Upadacitinib	Selective JAK1	Oral	2	Dose-depended improvement in EASI score; the 30-mg once-daily dose showed the greatest clinical benefit [170]	Upper respiratory tract infections
Arocitinib	Selective JAK1	Oral	2	Dose-depended improvement in IGA and EASI scores; rapid reduction in pruritus NRS scores by 2 days after the initiation of treatment [171]	Upper respiratory tract infection, headache, nausea, diarrhea
			3	Improvement in IGA, EASI-75, EASI-90, itch NRS and DLQI [172,173]	Nausea, nasopharyngitis, headache, atopic dermatitis
Gusacitinib	Pan-JAK (JAK1, JAK2, JAK3, TYK2) and SYK	Oral	1	Dose-dependent improvement in IGA, EASI-50, EASI-75 and pruritus NRS scores with highest efficacy for 40 mg dose [174]	No serious side effects
Cis-urocanic acid	Replacement therapy	Topical	1/2b	Improvement in EASI and IGA scores, reduce TEWL and cutaneous erythema [63]	No serious side effects
**L-histidine**	Replacement therapy	Oral	pilot	Improvement in EASI and SCORAD scores [64]	No serious side effects

### 5.4. Downregulation of FLG Expression by IL-17A

Although IL-17-producing Th17 cells have been proposed to play a potential role in AD, the pathogenic significance of IL-17A is not fully understood and conflicting results on this issue have been reported [69,182,183]. However, greater expression of Th17-related markers was observed in some phenotypes such as Asian, intrinsic, pediatric and elderly AD patients [54,105,183]. Thus, it is hypothesized that IL-17 targeting may have some benefit in selected populations. IL-17A binds two receptor complexes, IL-17RA/IL-17RC and IL—17RA/IL-17RD, in keratinocytes, activates the NF-κB and MAPKs and promotes keratinocyte proliferation and chemokine production [184]. IL-17A is reported to downregulate the expression of FLG via the C/CAAT-enhancer-binding proteins, particularly C/EBPB, which is another important transcription factor for IL-17A signaling [185,186].

Secukinumab, a selective anti-IL-17 inhibitor, was trialed in a 52-week phase 2 study in patients with moderate-to-severe AD. The results of this study, which integrated clinical assessments with extensive cellular and genomic biomarkers in skin, demonstrated that IL-17 is not a valid therapeutic target in patients with AD, including the subsets of patients with higher Th17 activation [187]. The results of another phase 2 trial investigating the efficacy of secukinumab in patients with moderate to severe AD (Secu_in_AD) are not available yet.

### 5.5. Downregulation of FLG Expression by IL-24

IL-24 is a multifunctional cytokine that belongs to the IL-20 family. Although enhanced expression of IL-24 is observed in the epidermis of model mice and in AD skin lesions, its implication in AD pathogenesis remains elusive [116]. However, it has been recently demonstrated that IL-24, acting alone, clearly decreases FLG expression. IL-24 is produced by Th2 lymphocytes and keratinocytes after stimulation with type 2 cytokines, such as IL-4, IL-13, and IL-31. IL-4/IL-13-mediated STAT6 activation upregulate the expression of periostin, which stimulates IL-24 production in keratinocytes. These results have highlighted the significance of IL-24 as a novel mediator of AD that seems to be a new therapeutic option [188]. On the other hand, IL-24 was previously determined to be a target cytokine of some oxidative AHR ligands [189,190]. Recently, Vu et al. have found that tapinarof, which upregulates FLG expression, also induced the secretion of IL-24. Notably, tapinarof alone restores the downregulation of FLG, however, inhibition of IL-24 or STAT3 by siRNA transfection augmented this effect. Those observations suggested that combined treatment with AHR modulator and JAK inhibitors may be a promising strategy for more effective AD therapy [191].

### 5.6. FLG Enhancement Therapy

A number of studies into identifying molecules with FLG-enhancing properties in vitro or in animal models have been performed. Otsuka et al. identified JTC-801, a selective antagonist for the nociception receptor that promoted FLG mRNA and protein expression in both human and murine keratinocytes. In addition, oral administration of JTC-801 promoted protein level of FLG and suppressed the development of AD-like skin inflammation in NC/Nga mice. The precise underlying mechanism remains unclear but the recently published data indicated that phosphoinositide 3-kinase (PIK3K)/protein kinase B (AKT) can be involved [192]. Drugs that activate the nuclear hormone receptors peroxisome proliferator-activated receptors (PPARs) have to be also been considered as potential agents for AD treatment. All tested topical PPAR-β/δ agonists (GW1514), PPAR-α agonists (clofibrate, and WY-14,643), and PPAR-γ agonists (ciglitazone and troglitazone) stimulate FLG production and keratinocyte differentiation and improve multiple parameters of the AD-like dermatosis in vitro and in mice models [193,194,195]. More recently, docosahexaenoic acid (DHA), a dual PPARα/γ agonist was shown to increase the expression of FLG and have anti-inflammation effects in monolayer keratinocytes and reconstructed human epidermis models [196]. Czarnowicki et al. reported also promising results for the liver X receptor (LXR) agonist. Topical VTP-38543 significantly increased FLG mRNA expression and reduced epidermal hyperplasia markers in patients with mild-to-moderate AD [197]. PPARs and LXR activators are likely to improve barrier function through at least 2 parallel mechanisms: stimulation of epidermal differentiation and lipid production [198]. Sirtuin 1 (SIRT1) a silent mating type information regulation 2 homolog is another promising target as SIRT1 has been found to promote FLG expression which suggests its critical role for skin barrier integrity [199,200]. Compared with normal human skin, SIRT1 is downregulated in both AD and non-AD lesions. Jin et al. found that adiponectin (Acrp30) treatment upregulated FLG expression in a dose-dependent and time-dependent manner through a SIRT1-mediated signal transduction pathway in human keratinocytes [201]. A recent study in mouse primary keratinocyte showed that treatment with the apolipoprotein B mRNA editing enzyme complex (APOBEC3) siRNA increases the expression levels of FLG and other keratinocyte differentiation markers [202]. Furthermore, widely used moisturizers, such as petrolatum, urea and glycerol, have been shown to alter the structure of the epidermis and increased the expression of key barrier differentiation proteins, including FLG, in patients with and without AD, in addition to reducing skin inflammation and inducing AMP expression [203,204,205].

Recently, the trend toward using medicines from nature as an alternative treatment for diseases, especially skin inflammation has been observed. An increasingly long line of evidence has indicated that certain herbal medicines can be helpful in skin disorders characterized by abnormalities in barrier function associated with reduced FLG levels. Topical apigenin, a naturally occurring flavonoid of plant origin, has been shown to upregulate FLG expression and significantly enhance permeability barrier homeostasis both in vivo and in vitro. Although the exact mechanisms are not clear, it is possible that the apigenin-induced FLG upregulation could result from its antioxidant properties [206]. A more recent study in a mice model of AD revealed that both apigenin and other biologically active compounds present in celery, apigetrin and luteolin have ameliorative effects on AD signs and symptoms [207]. Additionally, apigenin has been suggested to be a promising agent for itch-reducing therapy, through its modulation of the IL-31 cytokine [208]. Several components of a clinically proven Chinese medicinal pentaherbs formula, such as apigenin, quercetin, luteolin, ursolic acid and rosmarinic acid have been reported as the key compounds acting on crucial biological processes involved in AD, including inflammatory response, apoptosis, response to hypoxia and nitric oxide biosynthesis. [209]. Another study found that Urtica thunbergiana, a Korean traditional medicine treatment, recovers the FLG and protects the AD skin by decreasing scratching and epidermal thickness, as well as by promoting SC hydration [210].

Although the strategies presented above appear to be promising most of these candidate drugs still have not advanced beyond in vitro and in vivo models and their beneficial effect in human AD is yet to be determined.

## 6. Conclusions

Atopic dermatitis is one of the most common chronic inflammatory skin diseases with a high burden on patients’ quality of life, healthcare costs and morbidity. Currently, the systemic treatment of AD remains challenging and limited, as dupilumab is the only biologic approved for the treatment of moderate-to-severe AD in patients 6 years and older, with consistent long-term efficacy and safety trial data. Thus, the need for the development of novel treatment with increased efficacy and the potential for fewer systemic side effects is obvious. In recent years, many previously unknown details involved in the mechanisms of the pathogenesis of AD have been clarified and the new therapeutic approaches that target those specific pathophysiological pathways have been developed. It is becoming increasingly clear that skin barrier abnormalities and immune dysfunction are important aspects of AD pathology. However, FLG is a crucial epidermal protein that contributes to the skin barrier function and FLG mutations remain the most replicated and significant risk factor for AD. FLG deficiency which is influenced not only by the FLG genotype but also by inflammation and exogenous stressors is suggested to be a critical cause of AD. Since FLG is still placed at the very center of AD etiopathogenesis, therapeutic strategies to improve skin barrier function by restoring FLG deficiency are widely studied as a promising and beneficial treatment for AD. The gene-based and direct replacement FLG therapy is not currently available, thus, novel therapies enhancing FLG expression or blocking acquired FLG downregulation are a major target of many clinical trials with promising results. Although larger, well-designed, controlled studies are needed to evaluate the safety and efficacy of drugs mentioned in this paper and a few more at early stage need to be fully validated and in humans, the future holds the promise that restoration of FLG can be an important part of AD therapy.

## Figures and Tables

**Table 1:** Potential therapies targeting FLG expression.

Targets
Gene-based approach: “Read-through” drugs
Direct replacement of FLG
Indirect replacement therapy: Topical application of FLG metabolites: PCA, UCA L-histidine
Inhibiton of cytokine-mediated FLG downregulation: IL-4/IL-13 inhibitors IL-13 inhibitors JAK inhibitors IL-22 inhibitors IL-17 inhibitors IL-24 inhibitors
Enhancement FLG expression: AHR agonists JTC-801 Peroxisome proliferator-activated receptors (PPARs) agonists Liver X receptor (LXR) agonists Sirtuin 1 (SIRT1) Apolipoprotein B mRNA editing enzyme complex (APOBEC3) Petrolatum, urea, glycerol Herbal medicines: apigenin, quercetin, luteolin, ursolic acid, rosmarinic acid

**Table 2:** Key immunological pathways regulating FLG expression.

Pathway	Mechanisms Regulating FLG Expression	Effect on FLG Expression	Mechanisms of Targeted Therapy
**IL-4/IL13**	Upregulation of JAK/STAT signalling Downregulation of OVOL1 signalling Upregulation of periostin-IL-24 axis	Downregulation	Anti-IL4Rα mAb
**IL-13**	Upregulation of JAK/STAT signalling Downregulation of OVOL1 signalling Upregulation of periostin-IL24 axis	Downregulation	Anti-IL-13 mAb
**Janus kinase (JAK)**	Upregulation of STAT phosphorylation	Downregulation	JAK inhibitors
**IL-22**	Upregulation of JAK/STAT3 signalling Upregulation of IL-24	Downregulation	Anti-IL-22 mAb
**IL-17A**	Upregulation of C/CAAT-enhancer-binding proteins, particularly C/EBPB	Downregulation	Anti-IL-17A mAb
**IL-24**	Upregulation of JAK/STAT3 signalling	Downregulation	JAK inhibitors
